# Description of the karyotype of *Rhagomys rufescens* Thomas, 1886 (Rodentia, Sigmodontinae) from Southern Brazil Atlantic forest

**DOI:** 10.1590/S1415-47572010005000071

**Published:** 2010-09-01

**Authors:** André Filipe Testoni, Sérgio Luiz Althoff, André Paulo Nascimento, Francisco Steiner-Souza, Ives José Sbalqueiro

**Affiliations:** 1 Laboratório de Citogenética Animal, Departamento de Genética, Universidade Federal do Paraná, Curitiba, PRBrazil; 2 Laboratório de Biologia Animal, Departamento de Ciências Naturais, Fundação Universidade Regional de Blumenau, Blumenau, SCBrazil; 3 Laboratório de Genética, Departamento de Ciências Naturais, Fundação Universidade Regional de Blumenau, Blumenau, SCBrazil; 4 Programa de Pós-Graduação em Biologia Animal, Departamento de Zoologia, Universidade Federal do Rio Grande do Sul, Porto Alegre, RSBrazil; 5 Programa de Pós-Graduação em Genética, Departamento de Genética, Universidade Federal do Paraná, Curitiba, PRBrazil

**Keywords:** *Rhagomys rufescens*, Thomasomyini, Rodentia, Atlantic forest, karyotype

## Abstract

*Rhagomys rufescens* (Rodentia: Sigmodontinae) is an endemic species of the Atlantic forest from Southern and Southeastern Brazil. Some authors consider *Rhagomys* as part of the tribe Thomasomyini; but its phylogenetic relationships remain unclear. Chromosomal studies on eight specimens of *Rhagomys rufescens* revealed a diploid number of 2n = 36 and a number of autosome arms FN = 50. GTG, CBG and Ag-NOR banding and CMA_3_ /DAPI staining were performed on metaphase chromosomes. Eight biarmed and nine acrocentric pairs were found in the karyotype of this species. The X and Y chromosomes were both acrocentric. Most of the autosomes and the sex chromosomes showed positive C-bands in the pericentromeric region. The X chromosome showed an additional heterochromatic block in the proximal region of the long arm. Nucleolus organizer regions (NORs) were located in the pericentromeric region of three biarmed autosomes (pairs 4, 6 and 8) and in the telomeric region of the short arm of three acrocentrics (pairs 10, 12 and 17). CMA _3_ /DAPI staining produced fluorescent signals in many autosomes, especially in pairs 4, 6, and 8. This study presents cytogenetic data of *Rhagomys rufescens* for the first time.

The subfamily Sigmodontinae (Wagner 1843) comprises 74 genera and 377 species (Musser and Carleton, 2005) and includes predominantly South American Cricetidae rodents, such as *Rhagomys rufescens*. This species is endemic to the Atlantic forest from Southern and Southeastern Brazil, and has already been reported in Rio de Janeiro, Minas Gerais, São Paulo, Espírito Santo and Santa Catarina (Moojen, 1952; Emmons and Feer, 1997; Eisenberg and Redford, 1999; Nowak, 1999; Percequillo *et al.*, 2004; Pinheiro *et al.*, 2004; Metzger *et al.*, 2006; Pardini and Umetzu, 2006; Steiner-Souza *et al.*, 2008). The first record of *R. rufescens* in Southern Brazil was recently obtained at the *Parque Natural Municipal Nascentes do Garcia* (PNMNG), in the state of Santa Catarina (Steiner-Souza *et al.*, 2008).

*Rhagomys rufescens* was originally described as *Hesperomys rufescens*, based on a female collected in Rio de Janeiro, southeastern Brazil. In the beginning of the 20^th^ century, Thomas collected another specimen from an unknown locality, which was used for the description of the genus *Rhagomys* (Thomas 1917) (Percequillo *et al.*, 2004). A second species, *Rhagomys longilingua*, was recently described based on a male collected in the Montana forests in southern Peru (Luna and Patterson, 2003), that was later found to reach as far as Bolivia (Villalpando *et al.*, 2006).

*Rhagomys* is considered *incertae sedis* (Reig, 1980, 1984; McKenna and Bell, 1997; Smith and Patton, 1999; Musser and Carleton, 2005) or a “plesiomorphic Neotropical muroid”, according to Voss (1993) and Steppan (1995), and there is no consensus regarding its tribal position (Percequillo *et al.*, 2004). Nevertheless, some authors included *Rhagomys* in the tribe Thomasomyini based on morphological characters (Pacheco, 2003) or on nuclear IRBP gene sequences (D'Elia *et al.*, 2006, 2007). Cytogenetic studies on species of Thomasomyini have shown significant variation both in diploid number (2n = 20-82) and in the number of autosome arms (FN = 34-114) (Table 1). The species in Table 1 are grouped in “Andean” Thomasomyini, which includes genera with a predominantly Andean distribution (*sensu* Pacheco, 2003), and “Endemic Atlantic” Thomasomyini, an informal group named by Oliveira and Bonvicino (2002).

The objective of this study was to describe the karyotype of *Rhagomys rufescens* from southern Brazil after conventional and CMA_3_/DAPI staining, and GTG, CBG and Ag-NOR banding. The chromosomal data presented in this work can provide additional information for studies on both taxonomic and phylogenetic relationships.

Eight specimens (five males and three females) were analyzed. They were captured at PNMNG, at “Mono” locality (27°02'59” S, 49°08'57” W), in Indaial city, in the state of Santa Catarina, southern Brazil. This park is now part of Parque Nacional da Serra do Itajaí (PNSI) (Figure 1). The animals were caught in Sherman traps placed at 3 m from the ground, according to Kierulff *et al.* (1991), with adaptations.

Chromosomes were obtained directly from bone marrow according to the method of Ford and Hamerton (1956), modified by Sbalqueiro and Nascimento (1996). Conventional Giemsa staining (5%) was used to determine diploid number (2n), chromosomal morphology and the number of autosomal arms (FN). At least 20 metaphase plates per individual were examined. GTG, CBG and Ag-NOR banding were performed according to Seabright (1971), Sumner (1972), and Howell and Black (1980), respectively. Chromomycin A3 (CMA_3_), and 4,6-diamidino-2-phenylindole (DAPI) were used according to Schweizer (1976). Chromosomes were classified as metacentric (M), submetacentric (SM), and acrocentric (A).

Skins and skulls of specimens were deposited at the Coleção Zoológica da Fundação Universidade Regional de Blumenau (CZFURB), in Blumenau, State of Santa Catarina, Brazil.

Analyses after conventional staining showed 2n = 36 and FN = 50 in all specimens (Figure 2a), with five metacentric pairs (1, 3, 6 and 8), three submetacentric (pairs 2, 4 and 5) and nine acrocentric pairs (pairs 9 to 17), decreasing gradually in size. The X chromosomes were acrocentric, indistinguishable from pair 9, whereas the Y chromosome was also acrocentric and similar in size to pair 10. All chromosome pairs, including the sex chromosomes, could be identified after G-banding. The X chromosome showed two positive bands in the medium portion of the long arm and the Y chromosome had one proximal band in the long arm (Figure 2b).

C-banding revealed pericentromeric constitutive heterochromatic blocks in most autosomes and also in the sex chromosomes. An additional interstitial C-band was present in the proximal region of the long arm of the X chromosome (Figure 2c).

NORs were detected in the pericentromeric region of pairs 4, 6 and 8, and in the telomeric region of the short arm of acrocentric pairs 10, 12 and 17 (Figure 2d). Two to twelve NORs were observed, with a mean of 7.33 ± 3.19 per cell (N = 39).

**Figure 1:**
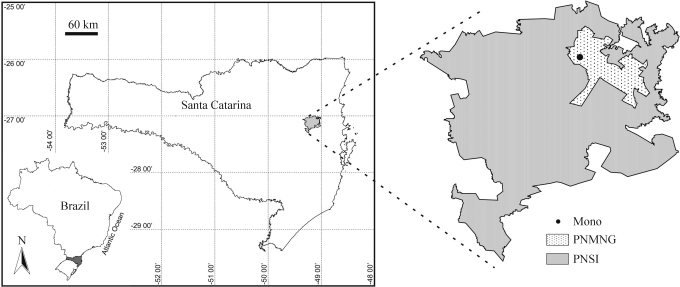
“Mono” locality, data collection site of specimens at Parque Natural Municipal Nascentes do Garcia (PNMNG), part of Parque Nacional Serra do Itajaí (PNSI), state of Santa Catarina, Southern Brazil.

**Figure 2:**
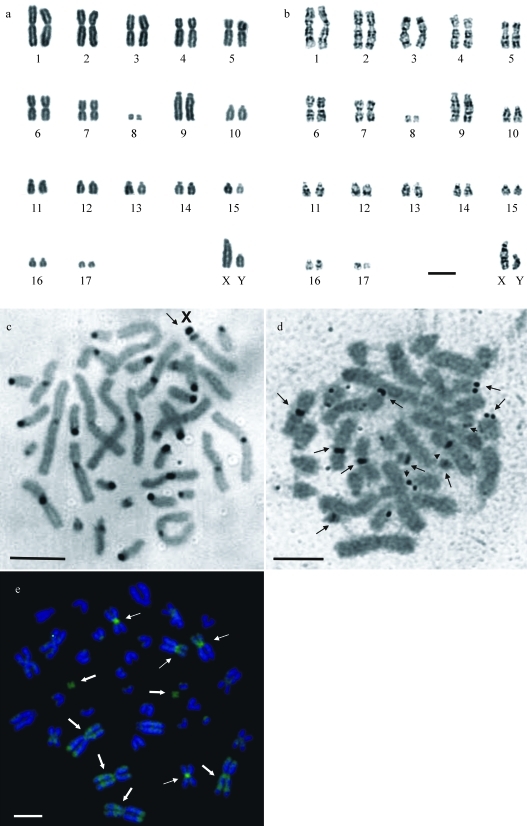
Karyotype of *Rhagomys rufescens* (male) after (a) conventional staining and (b) G-banding. Bar = 2,5 μm. (c) Metaphase of *Rhagomys rufescens* after C-banding. The arrow points to the X chromosome. (d) Metaphase after Ag-NOR staining. The arrows point to the nucleolus organizing regions. (e) Metaphase after CMA_3_/DAPI staining. The arrows point to the chromosomes with intense fluorescent CMA_3_ signals in the “p” and “q” arms (thick arrow) and pericentromeric region (thin arrow).

The double staining with the GC- and AT-specific fluorochromes, CMA_3_ and DAPI, respectively, showed intense fluorescent CMA_3_ signals in the pericentromeric region of pairs 4 and 6, and throughout the length of pair 8. Less intense signals were observed in other pairs (Figure 2e).

*Rhagomys* is a polytypic genus composed by *R. longilingua* and *R. rufescens*. After comparative morphological analyses, Pacheco (2003) considered it monophyletic, although the two forms show a discontinuous distribution: *R. longilingua* can be found in Peru and Bolivia, whereas *R. rufescens* occurs in southern and southeastern Brazil. The phylogenetic relationships of this genus with other Sigmodontinae are controversial and uncertain and it has been previously included in different tribes of this subfamily.

Pacheco (2003) compared morphological characters of *R. rufescens* to those of various species of Sigmodontinae. This author suggested a phylogenetic relationship with the tribe Thomasomyini: *Abrawayaomys*, *Aepeomys*, *Chilomys*, *Delomys*, *Juliomys*, *Phaenomys*, *Rhipidomys*, *Thomasomys* (including *Erioryzomys* and *Inomys*), *Wiedomys,* and *Wilfredomys*. *R. rufescens* appeared as a sister group of *Abrawayaomys ruschii* or within the *“*Andean” Thomasomyine group (*Thomasomys*, *Aepeomys*, *Chilomys* and *Rhipidomys*).

After analyses of the nuclear IRBP (Interphotoreceptor Retinoid Binding Protein) gene sequences, D'Elia *et al.* (2006) suggested grouping *Rhagomys longilingua* with the Thomasomyini species as a sister-group of *Thomasomys* and as part of a larger clade that also includes *Aepeomys* and *Rhipidomys*.

On the other hand, Percequillo *et al.* (2004), based on mitochondrial cytochrome B sequences, concluded that the position of *R. rufescens* within Sigmodontinae was uncertain and that *Rhagomys* was either closely associated to *Andinomys*, followed by a *Thomasomys*-*Rhipidomys* group, or closer to *Juliomys*, followed by *Andinomys*.

Therefore, all these studies suggested a phylogenetic relationship of *Rhagomys* with Thomasomyini species. So far, cytogenetic data have shown a significant variation in both diploid number (2n = 20-82) and FN (34-114) (Zanchin *et al.*, 1992b Bonvicino and Geise, 1995; Bonvicino and Otazu, 1999; Oliveira and Bonvicino, 2002; Costa *et al.*, 2007). Nevertheless, most species presented 2n = 44 and a predominance of acrocentric chromosomes, which were the cases of the species of *Rhipidomys* and *Thomasomys* (Table 1), possibly a sister group of *Rhagomys* (Pacheco, 2003; Percequillo *et al.*, 2004, D'Elia *et al.*, 2006). These results differ from our chromosome data of *Rhagomys rufescens* (2n = 36 and FN = 50), which had eight biarmed and nine acrocentric autosomal pairs.

The cytogenetic data of *Rhagomys rufescens* (2n = 36 and FN = 50) described herein are the first for this genus. Pair 9 and the X chromosome were undistinguishable after conventional staining because of their similar sizes and morphologies. However, GTG and CBG banding patterns showed significant differences allowing their individual identification. The two interstitial G-bands in the long arm of the X chromosome, characteristic of mammalian X chromosomes (Pathack and Stock, 1974), could be observed. Furthermore, an additional block of interstitial constitutive heterochromatin was present in the proximal region of the long arm of X chromosome, whereas pair 9 only presented a pericentromeric heterochromatic block. The Y chromosome, which is almost completely heterochromatic in many species of South-American rodents (Sbalqueiro *et al.* 1991; Andrades-Miranda *et al.*, 2001), only presented a positive C-band in the pericentromeric region in *Rhagomys rufescens*.

After double fluorochrome staining, CMA3-positive and DAPI-negative signals were present in sites coincident with all AgNORs. The correlation of NORs with GC-rich sites is relatively common among vertebrates (Schmid, 1982; Amemiya and Gold, 1986; Artoni *et al.*, 1999, among others), although the reverse correlation is not always valid. Additional GC-rich sites were also observed, mainly in the first three chromosome pairs. These sites were euchromatic domains adjacent to G-bands, known to correspond to GC-rich isochores (R-bands), especially close to the telomeric region (Bernardi, 1993; Holmquist and Ashley, 2006). However, several authors suggested the use of the silver staining technique in conjunction with FISH (rDNA probes) to confirm the number and location of NORs (Santos *et al.*, 2001; Fagundes *et al.*, 2003; Leite-Silva *et al.*, 2003).

The comparison of the chromosome data presented herein to those of the other Thomasomyini species mentioned above does not allow to determine the taxonomic relationship of *Rhagomys rufescens.* The scarcity of cytogenetic data of a larger number of species and the lack of techniques that could show more details about chromosome structure makes further taxonomic analysis a difficult task. It is evident that several chromosome rearrangements have contributed to the karyotypic variability observed in Thomasomyini. Complementary data obtained from differential staining associated with FISH techniques, such as ZOO-FISH (Hass *et al.*, 2008), is necessary for clarifying the mechanisms of karyotypic evolution in this group, and hence contribute to determine the taxonomic position of this genus. The data reported herein are important as a first characterization of the chromosome complement of *R. rufescens* because it allows the identification of some primary features of its karyotype.

## Figures and Tables

**Table 1:** Diploid numbers (2n) and number of autosome arms (FN) of Thomasomyini species.

Species	2n	FN	Authors
“Andean” Thomasomyini species			
*Aepeomys fuscatus*	54	62	Gardner and Patton (1976)
*Aepeomyslugens*	28	48	Aguilera *et al.* (2000)
*Aepeomyslugens*	44	46	Gómez-Laverde *et al.* (1997)
*Aepeomys* sp.	44	46	Aguilera *et al.* (1994)
*Aepeomysreigi*	44	46	Ochoa *et al.* (2001)
*Rhipidomys cearanus*	44	-	Zanchin *et al.* (1992a)
*Rhipidomys latimanus*	44	48	Gardner and Patton (1976)
*Rhipidomys leucodactylus*	44	48	Zanchin *et al.* (1992a)
*Rhipidomys leucodactylus*	44	48	Andrades-Miranda *et al.* (2002)
*Rhipidomys leucodactylus* cytotype 1	44	52	Andrades-Miranda *et al.* (2002)
*Rhipidomys mastacalis*	44	74	Zanchin *et al.* (1992a)
*Rhipidomys mastacalis* cytotype 1	44	80	Andrades-Miranda *et al.* (2002)
*Rhipidomys mastacalis* cytotype 2	44	76	Andrades-Miranda *et al.* (2002)
*Ripidomys* cf. *mastacalis*	44	52	Silva and Yonenaga-Yassuda (1999)
*Rhipidomys nitela*	48	68	Andrades-Miranda *et al.* (2002)
*Rhipidomys sclateri*	44	48	Aguilera *et al.* (1994)
*Rhipidomys* sp.	44	48	Svartman and Almeida (1993)
*Rhipidomys* sp.	44	49	Svartman and Almeida (1993)
*Rhipidomys* sp.	44	50	Zanchin *et al.* (1992a)
*Rhipidomys* sp. A	44	61	Silva and Yonenaga-Yassuda (1999)
*Rhipidomys* sp. B	50	71,72	Silva and Yonenaga-Yassuda (1999)
*Thomasomys andersoni*	44	42	Salazar-Bravo and Yates (2007)
*Thomasomys aureus*	44	42	Gardner and Patton (1976)
*Thomasomys kalinowskii*	44	44	Gardner and Patton (1976)
*Thomasomys laniger*	42 40	40 40	Aguilera *et al.* (2000) Gómez-Laverde *et al.* (1997)
*Thomasomys monochromos*	42	42	Gardner and Patton (1976)
*Thomasomys niveipes*	24	42	Gomez-Laverde *et al.* (1997)
*Thomasomys notatus*	44	44	Gardner and Patton (1976)
*Thomasomys* sp.	44	42	Gardner and Patton (1976)
*Thomasomys taczanowskii*	44	44	Gardner and Patton (1976)
*Thomasomys vestitus*	44	42	Aguilera *et al.* (2000)
“Endemic Atlantic” Thomasomyini species			
*Delomys collinus*	82	86	Bonvicino and Geise (1995)
*Delomys dorsalis*	82	80	Zanchin *et al.* (1992b)
*Delomys sublineatus*	72	90	Zanchin *et al.* (1992b)
*Phaenomys ferrugineus*	78	114	Bonvicino *et al.* (2001)
*Juliomys ossitenuis*	20	36	Costa *et al.* (2007)
*Juliomys pictipes*	36	34	Bonvicino and Otazu (1999)
*Juliomys rimofrons*	20	34	Oliveira and Bonvicino (2002)
*Juliomys* sp.	32	48	Paresque *et al.* (2009)
“Other” Thomasomyini species			
*Abrawayaomys ruschii*	58	-	Pereira *et al.* (2008)
*Andinomy edax*	56	56	Spotorno *et al.* (2001)
*Irenomys tarsalis*	64	98	Ojeda *et al.* (2004)
*Rhagomys rufescens*	36	50	Present report
*Wiedomys cerradensis*	60	88	Gonçalves *et al.* (2005)
*Wiedomys pyrrhorhinos*	62	86	Maia and Langguth (1981)
